# Effect of Substrate and Thickness on the Photoconductivity of Nanoparticle Titanium Dioxide Thin Film Vacuum Ultraviolet Photoconductive Detector

**DOI:** 10.3390/nano12010010

**Published:** 2021-12-21

**Authors:** Marilou Cadatal-Raduban, Tomoki Kato, Yusuke Horiuchi, Jiří Olejníček, Michal Kohout, Kohei Yamanoi, Shingo Ono

**Affiliations:** 1 Centre for Theoretical Chemistry and Physics, School of Natural and Computational Sciences, Massey University, Auckland 0632, New Zealand; 2 Institute of Laser Engineering, Osaka University, 2-6 Yamadaoka, Suita 565-0871, Osaka, Japan; yamanoi-k@ile.osaka-u.ac.jp; 3 Department of Physical Science and Engineering, Nagoya Institute of Technology, Nagoya 466-8555, Aichi, Japan; tomoki.kato.onolab@gmail.com (T.K.); masa.horiuchi09@gmail.com (Y.H.); ono.shingo@nitech.ac.jp (S.O.); 4 Department of Low-Temperature Plasma, Institute of Physics, Czech Academy of Sciences, Na Slovance 2, 182 21 Prague, Czech Republic; olejn@fzu.cz (J.O.); kohout@fzu.cz (M.K.)

**Keywords:** nanoparticle, thin film, titanium dioxide, wide band gap, semiconductor, vacuum ultraviolet, photoconductive detector

## Abstract

Vacuum ultraviolet radiation (VUV, from 100 nm to 200 nm wavelength) is indispensable in many applications, but its detection is still challenging. We report the development of a VUV photoconductive detector, based on titanium dioxide (TiO_2_) nanoparticle thin films. The effect of crystallinity, optical quality, and crystallite size due to film thickness (80 nm, 500 nm, 1000 nm) and type of substrate (silicon Si, quartz SiO_2_, soda lime glass SLG) was investigated to explore ways of enhancing the photoconductivity of the detector. The TiO_2_ film deposited on SiO_2_ substrate with a film thickness of 80 nm exhibited the best photoconductivity, with a photocurrent of 5.35 milli-Amperes and a photosensitivity of 99.99% for a bias voltage of 70 V. The wavelength response of the detector can be adjusted by changing the thickness of the film as the cut-off shifts to a longer wavelength, as the film becomes thicker. The response time of the TiO_2_ detector is about 5.8 μs and is comparable to the 5.4 μs response time of a diamond UV sensor. The development of the TiO_2_ nanoparticle thin film detector is expected to contribute to the enhancement of the use of VUV radiation in an increasing number of important technological and scientific applications.

## 1. Introduction

High energy radiation in the vacuum ultraviolet (VUV) region that spans the wavelength range from 200 nm down to 100 nm is indispensable in numerous technological applications, such as surface treatment, photochemical processing, optical cleaning of semiconductor substrates, and sterilization of medical apparatus; in addition, it is crucial for scientific research, primarily in gas chromatography and molecular spectroscopy, for example, to probe carbon–carbon and nitrogen–nitrogen triple bonds in alkynes. The applications of VUV light sources are increasing owing to its higher photon energy compared with the ultraviolet (UV) region, from 400 nm down to 200 nm. Alongside the increasing importance of VUV radiation in various applications, there has also been rapid development of VUV light sources [1,2,3]. As such, development of detectors in the VUV region is also crucial. Various reports focused on the detection of VUV light through scintillation in rare earth-doped wide band gap insulators [4,5,6] and wide band gap semiconductors [7,8,9]. Scintillation relies on the excitation and de-excitation of an activator ion (in rare earth-doped insulators), or the generation and recombination of electron and hole pairs (in wide band gap semiconductors), that result in photoluminescence (PL) emission, usually in the longer ultraviolet or visible wavelength regions, followed by the detection of this PL emission using a conventional detector, such as a photomultiplier tube. With this method, the scintillation process needs to be optimized so that the PL emission is intense enough to be detected reliably. Photoconduction is another approach in detecting VUV radiation [10,11]. In this method, electron–hole pairs (also called photo-generated carriers) are created when the wide band gap material absorbs an incoming photon. The movement of these photo-generated carriers in the presence of an external electric field produces a photocurrent, which characterizes the photoconductive detector and enables the direct measurement of the VUV radiation.

Titanium dioxide (TiO_2_) is a wide band gap photosensitive semiconductor that has excellent chemical, physical, and optical properties. TiO_2_ is a well-known photocatalyst, but it was also reported as a UV sensor in the 235 nm to 420 nm wavelength range [12] along with gallium nitride (GaN) [13], aluminum nitride (AlN) [14], and zinc oxide (ZnO) [15]. Diamond has been explored for VUV detection but photodetection requires diamond films to have sufficient low defect and concentration of n-type and p-type dopants, in addition to the high cost of producing high-quality diamonds [16,17,18]. III–V nitrides, such as cubic boron nitride (c-BN), have better photoconductivity characteristics, including easier p- and n-type doping, compared with diamond, but its photoconductivity is hampered by the poor quality of materials [19,20]. Recently, AlN [21], amorphous boron nitride [22], hexagonal boron nitride (h-BN) [23,24], and magnesium oxide (MgO) [25] have also attracted attention as potential VUV photodetectors. Among these wide band gap semiconductor materials, fabrication of TiO_2_ is relatively easier and cheaper. In this work, we harness the excellent properties of TiO_2_ to demonstrate the potential of TiO_2_ thin films as room-temperature VUV photoconductive detectors. Furthermore, we also investigate the improvement of its photoconductivity in the VUV region using TiO_2_ thin films deposited on high resistivity undoped silicon (Si), quartz glass (SiO_2_), and soda lime glass (SLG) substrates with thicknesses of 80 nm, 500 nm, and 1000 nm. Our results show that TiO_2_ thin films in general—but especially TiO_2_ thin films deposited on SiO_2_ substrate with a thickness of 80 nm exhibit higher photoconductivity, with photocurrents reaching the milli-Ampere range, compared with our previous works using wide band gap fluorine-based insulator thin film photoconductive detectors [26,27] and gamma-ray irradiated detectors, based on amorphous TiO_2_ on SLG substrate [28]. The potential of using TiO_2_ thin films as photoconductive detectors of VUV radiation will contribute to the enhancement of the many applications of this high energy radiation.

## 2. Materials and Methods

Reactive direct current magnetron sputtering was used to deposit the TiO_2_ thin films on the surface of 3 unheated substrates: high resistivity undoped Si ((1 0 0) plane orientation, 1 kΩ·cm resistivity, 0.795 ± 0.005 mm thickness), SiO_2_ glass, and SLG microscope slides (Menzel-Gläser, Thermo Scientific, Braunschweig, Germany). The target used was pure titanium (99.995% purity) with a diameter of 15 cm and thickness of 6 mm. The chamber was evacuated to a base pressure of 1 × 10^−3^ Pa before deposition. The films were then sputtered in reactive argon (Ar) and oxygen (O_2_) atmosphere under a total gas pressure of 1.3 Pa. The ratio of the Ar:O_2_ gas mixture was 4:1, which was achieved by maintaining the flow rate of Ar at 20 sccm and O_2_ at 5 sccm. The absorbed power was 600 W, corresponding to a power density of 3.4 W/cm^2^. Under such conditions, the average deposition rate was 5 nm/min. The deposition time was adjusted to achieve a deposited film thickness of 80 nm, 500 nm, and 1000 nm. The thin films were then annealed in air for 8 h at 450 °C. After annealing, the thickness of the films was confirmed to an accuracy of less than ±10% by surface profilometry (Alpha Step 500, Tencor, Santa Clara, CA, USA). To fabricate the photoconductive detector, a pair of interdigitated aluminum (Al) electrodes were deposited on the surface of each of the films using the same chamber as for the TiO_2_ thin film deposition. The electrodes were deposited through a stainless steel mask using a pure Al target (99.99% purity) in an Ar atmosphere. The working pressure during deposition was 1 Pa and the Ar flow rate was 20 sccm. The thickness of the electrodes was 500 nm. The distance between the electrodes was 0.2 mm. The length of the electrodes was 7.8 mm. The patterned area with the electrodes provided a sensing area of about 75 mm^2^.

The crystallinity of the TiO_2_ thin film detectors were determined using an Empyrean X-ray diffractometer in grazing incidence geometry (GIXRD) with a Cu Kα radiation (λ = 0.154 nm) and 2θ range from 10° to 90° in a step size of 0.01°. Qualitative analyses of the diffraction patterns were performed using a HighScore Plus 4.0 software package (PANanalytical, Cambridge, UK). The optical properties of the detectors were evaluated by obtaining their transmission spectra from 250 nm to 750 nm using a double-beam UV-visible–near infrared spectrophotometer (HITACHI U-4100, Tokyo, Japan). The time-independent and time-resolved photoluminescence (PL) spectra of each thin film was obtained by exciting the thin film with the frequency-tripled emission (3ω, 290 nm) of a mode-locked Ti:sapphire laser system (Spectra-Physics Tsunami Oscillator and Spitfire Regenerative Amplifier) operating at 1 kHz repetition rate and 100 fs pulse duration. The PL emission from the thin film was focused onto the entrance slit of a spectrograph coupled to a streak camera and a charge-coupled device (CCD) camera.

The photoconductivity of the thin film detectors was evaluated by illuminating their surface with a VUV lamp (Hamamatsu Photonics, Hamamatsu, Japan) emitting at a peak wavelength of 160 nm [28] and a radiant intensity of about 1.4 μW cm^−2^ mm^−1^ at a distance of 50 cm from the lamp. While applying a bias voltage of up to 100 V across the Al electrodes, the photocurrent produced by the photo-generated carriers as they drifted towards the Al electrodes was measured using an ultra-high-resistance electrometer (ADCMT 8340A, Hamamatsu, Japan). The dark current was also measured using the same set-up but with the VUV lamp turned off. The wavelength response of the detectors was evaluated by applying a bias voltage of 10 V while illuminating the detectors with a D_2_ lamp (Hamamatsu Photonics K.K., Shizuoka, Japan), emitting from 400 nm down to 115 nm wavelengths, coupled with a vacuum ultraviolet electroscope (BUNKOUKEIKI Co., Ltd. Kv 200, Tokyo, Japan). The vacuum level during VUV measurements was maintained at around 250 Pa. The time response of the detectors was determined using a VUV flash lamp emitting at 170 nm wavelength (USHIO Inc., Japan VUV Aligner SUS740 with a VUV lamp of UWSFL-250303V). The waveform of the time response was obtained by recording the photocurrent using an oscilloscope after an amplification circuit. The time response profiles were processed by applying a 2.5 MHz low-pass filter using fast Fourier transform analysis. All measurements were performed at room temperature.

## 3. Results and Discussion

### 3.1. TiO_2_ Photoconductive Detector with 80 nm Film Thickness on Si, SiO_2_, and SLG Substrates

**Figure 1:**
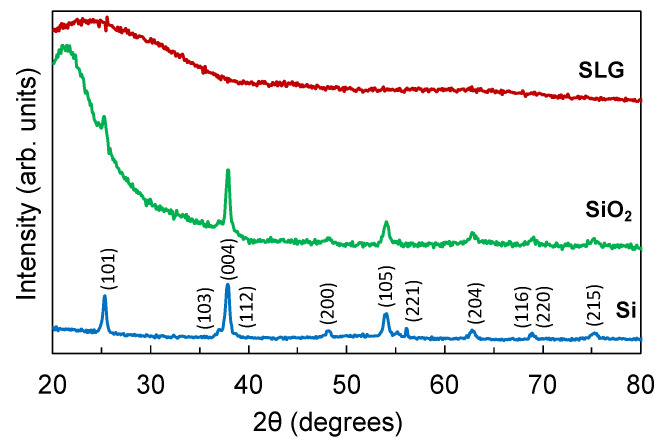
GIXRD spectra of TiO_2_ films deposited on Si, SiO_2_, and SLG substrates with a film thickness of 80 nm.

Figure 1 shows the GIXRD spectra of the TiO_2_ thin films deposited on Si, SiO_2_, and SLG substrates. The films have a thickness of 80 nm. The film deposited on the SLG substrate is amorphous while the films deposited on the Si and SiO_2_ substrates are crystalline. The diffraction peaks in the crystalline films can be indexed to the anatase phase of TiO_2_. The films on both Si and SiO_2_ substrates have a preferred grain orientation in the (004) direction at about 37.80°. The average crystallite size was determined using the Schrerrer formula, as follows:

(1) $$D=\frac{0.89\lambda }{B\cos\theta }$$

where *λ* is the 0.154 nm X-ray wavelength used during XRD measurement, *θ* is the diffraction angle of the peaks, and *B* is the full width at half maximum (FWHM) of the peaks. The film on the Si and SiO_2_ substrates have an average crystallite size of 5.1 nm and 11 nm, respectively. We note that thinner films (50 nm thickness) were also fabricated but these were all amorphous even after annealing.

**Figure 2:**
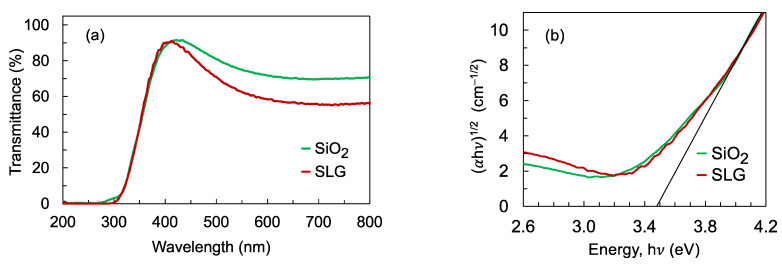
(**a**) Transmission spectra of the films deposited with a thickness of 80 nm on SiO_2_ and SLG substrates; (**b**) Tauc plots showing a similar optical band gap energy of 3.48 eV for the films deposited on both SiO_2_ and SLG substrates. The optical band gap was estimated from the x-intercept of the linear region.

The transmission spectra of the films deposited with a thickness of 80 nm on SiO_2_ and SLG substrates are shown in Figure 2a. The contribution from the substrate was excluded by deconvolving the transmission spectrum of the film from the transmission spectrum of a reference substrate. The Tauc plots in Figure 2b show that the films have similar optical band gaps at around 3.48 eV but the transmission spectrum shows that the transparency of the films is seen to decrease from around 430 nm wavelength, with a larger decrease in the transparency being observed for the film deposited on the SLG substrate. The decrease in transmittance implies that defects that could affect the detector’s photoconductivity exist in the film.

**Figure 3:**
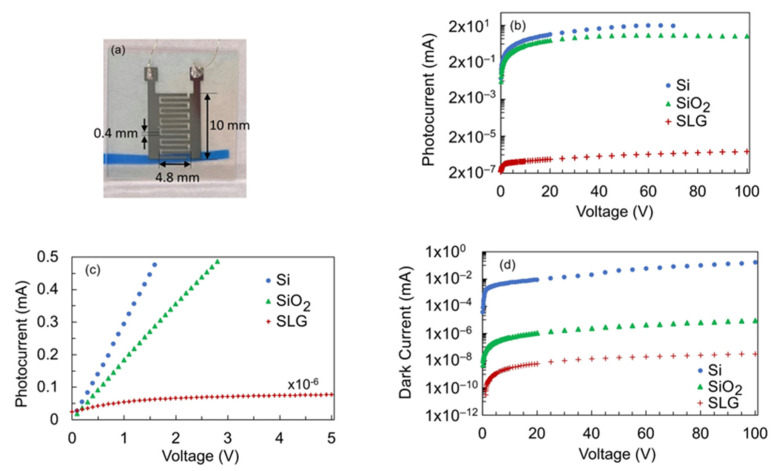
(**a**) Photograph of the VUV photoconductive detector with the Al electrodes on the surface of the TiO_2_ thin film; (**b**) I–V characteristics of the detectors under VUV illumination. The missing data points for the photocurrent of the detector on Si substrate when the bias voltage was more than 70 V is due to the photocurrent exceeding the detection limit of the electrometer; (**c**) linear plot of the photocurrent at low bias voltage to emphasize the nonlinear behavior of the photocurrent from the film detector on SLG substrate; (**d**) I–V characteristics of the detectors under darkness when the VUV lamp is turned off.

Figure 3a shows a representative photograph of the TiO_2_ photoconductive detector with the Al electrodes on the surface of the TiO_2_ thin film. In this photograph, the film was deposited on the SiO_2_ substrate. The current–voltage (I–V) characteristics of the detectors under VUV illumination are shown in Figure 3b. The detector that used SLG as substrate exhibited the smallest photocurrent, with a value of 3.00 × 10^−6^ mA at 70 V bias voltage. On the other hand, the detector with the Si and SiO_2_ substrates exhibited between 6 and 7 orders of magnitude higher photocurrent. The photocurrent of the TiO_2_ detector on SiO_2_ and Si substrates were 5.35 mA and 19.07 mA, respectively, at a bias voltage of 70 V. The photocurrent of the detector on Si substrate could potentially be higher at 100 V bias voltage as the photocurrent exceeded the detection limit of the electrometer when the bias voltage was increased beyond 70 V. The photocurrent of a blank Si substrate was measured for reference and was found to be two orders of magnitude smaller compared with the photocurrent of the TiO_2_ film deposited on the Si substrate (see Figure S1). Moreover, the photocurrent and dark current values from the Si substrate were similar. These confirm that the Si substrate is not photoconductive.

**Figure 4:**
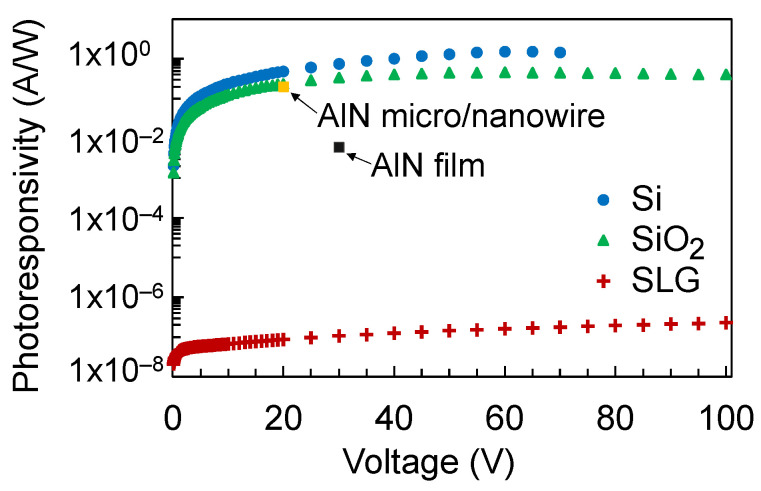
Photoresponsivity of TiO_2_ films deposited on Si, SiO_2_, and SLG substrates at 70 V bias voltage. The photosensitivity of an AlN micro/nanowire and AlN film from previous reports [10] are also shown for comparison.

The photoresponsivity (*R_λ_*) of the detectors was estimated using the following:

(2) $$R_{\lambda }=\frac{I_{photo}}{P_{in}},$$

where *I_photo_* is the photocurrent and *P_in_* is the input optical power which is estimated to be 13.2 mW at the wavelength *λ* = 160 nm. The photoresponsivity is shown in Figure 4. The photoresponsivity of the detector on Si, SiO_2_, and SLG substrates at 70 V bias voltage are 1.45 A/W, 0.44 A/W, and 1.79 × 10^−7^ A/W, respectively, as summarized in Table 1. In comparison with previously reported VUV photodetectors, the photoresponsivity of AlN micro/nanowire was reported to be 0.2 A/W at 20 V bias voltage and 160 nm wavelength while the photoresponsivity of AlN film was reported to be 0.006 A/W at 30 V bias voltage and 160 nm wavelength [10]. At 20 V bias voltage, the photoresponsivity of the TiO_2_ detector on Si and SiO_2_ substrates are 0.48 A/W and 0.23 A/W, respectively. At 30 V bias voltage, the photoresponsivity of the TiO_2_ detector on Si and SiO_2_ substrates are 0.74 A/W and 0.34 A/W, respectively.

Figure 3c shows a zoomed-in version of the photoconductivity shown in Figure 3b, focusing on the low bias voltage. The photocurrent was plotted using a linear scale to emphasize the nonlinear behavior of the photocurrent from the detector on the SLG substrate, while the detectors on the Si and SiO_2_ substrates exhibited a rapid linear increase in the photocurrent. The nonlinear photocurrent implies reduced photo-generated carrier mobility, which could be due to the amorphous nature of the film on SLG as well as the existence of defects that manifested in the decrease in the film’s transparency from around 430 nm wavelength (Figure 2). Defects also serve as trapping centers for the photo-generated carriers, thereby reducing the carrier density in the detector on SLG. The reduced carrier density and mobility would be responsible for the lower photocurrent observed from the detector on the SLG substrate compared with the detectors on the Si and SiO_2_ substrates. Looking at the dark current in Figure 3d, the detector on the SLG substrate exhibited the lowest dark current with a value of 3.03 × 10^−8^ mA at a bias voltage of 70 V. The dark current from the detector on Si substrate was the highest with a dark current value of 0.15 mA at 70 V bias voltage. The dark current from the detector on SiO_2_ substrate is 8.83 × 10^−6^ mA at 70 V bias voltage. To consider the photocurrent and dark current in the overall performance of the detectors, the photosensitivity of the detectors was calculated using the following formula:

(3) $$S=\frac{I_{photo}-I_{dark}}{I_{dark}}$$

where $I_{photo}$ is the photocurrent when the detector is under illumination and $I_{dark}$ is the dark current when the detector is under darkness [29]. The calculation results for a bias voltage of 70 V are summarized in Table 1. It can be inferred that, although the detector on Si substrate exhibited the highest photocurrent, its dark current is 5–7 orders of magnitude higher compared with the dark current of the other two detectors. As a result, the detector on SiO_2_ substrate showed the highest photosensitivity. As mentioned earlier, the average crystallite size of the TiO_2_ film on Si substrate is about half the size of the film on SiO_2_ substrate (5.1 nm vs. 11 nm). The smaller crystallite size indicates that there are more boundaries, which contribute to the adsorption of oxygen onto the TiO_2_ crystallite interfaces, as well as increased Ti interstitials. The increased oxygen vacancies and interstitial Ti ions resulted to the large dark current in the detector on Si substrate. A similar phenomenon was observed in the ZnO-based UV detector [30]. Consequently, the large dark current decreased the photosensitivity of the detector on Si. These results demonstrate that the detector’s photoconductivity is influenced by the combined effect of the film’s crystallinity, optical quality, and crystallite size.

**Figure 5:**
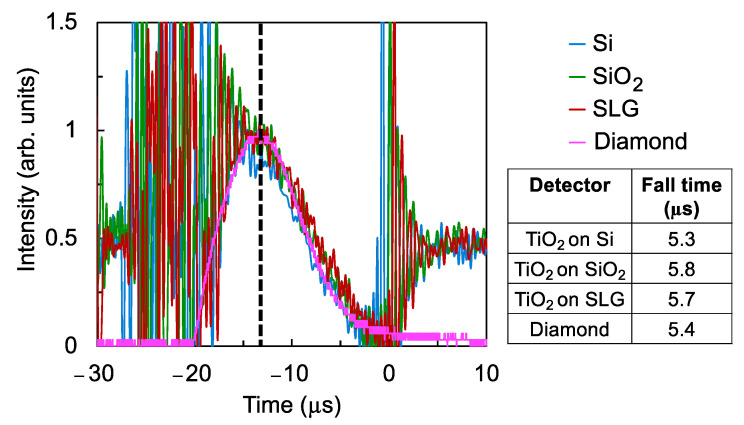
Time response of the TiO_2_ thin film detectors. For comparison, the time response of a commercial diamond UV sensor is also shown.

The time response of the detectors was measured and shown in Figure 5. For comparison, the time response of a commercially available diamond UV sensor (Hamamatsu H8496-26) was also measured. The noise that is observed from around −30 to −15 μs and from 0 to 10 μs is caused by the VUV flash lamp. To evaluate the response speed of the TiO_2_ thin film detectors, the fall time was estimated from the time it takes for the intensity value of the time response waveform to decrease to 50%. The detector on Si substrate exhibited a faster response time compared with those on SiO_2_ and SLG substrates, while it was comparable to the response time of the diamond sensor. The smaller crystallite sizes on the film on Si contribute to more crystallite boundaries and therefore a shorter carrier lifetime as the mean free path of the moving carriers would also be shorter [29]. As a result, the photocurrent decays faster, resulting in a faster response speed.

### 3.2. Photoconductive Detector with 80 nm, 500 nm, and 1000 nm TiO_2_ Film Thickness on SiO_2_ Substrate

**Figure 6:**
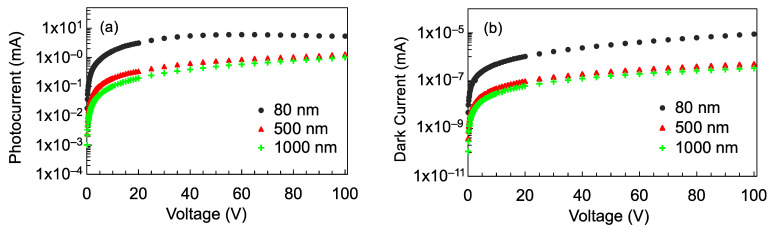
(**a**) I–V characteristics of the TiO_2_ detectors under VUV illumination; (**b**) I–V characteristics of the detectors under darkness.

**Figure 7:**
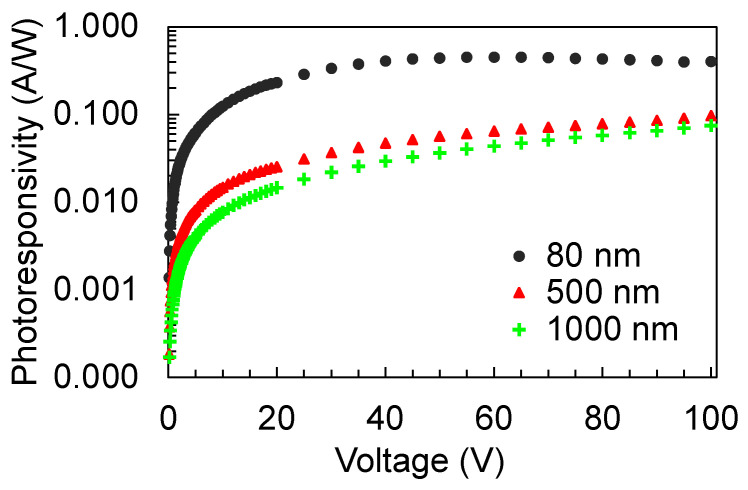
Photoresponsivity of the detectors with 80 nm, 500 nm, and 1000 nm thick TiO_2_ film on SiO_2_ substrate.

Based on the preceding results, where the TiO_2_ detector on the SiO_2_ substrate showed the best photosensitivity, the photoconductivity of this detector for different TiO_2_ film thicknesses was explored further. Figure 6a,b show the I–V characteristics of the detectors under illumination and under darkness, respectively. Interestingly, the highest photocurrent was obtained for the 80 nm film thickness and as the thickness increased, the photocurrent also decreased. The dark current also appears to be larger for the 80 nm film thickness. The photoresponsivity of the detectors are shown in Figure 7. As the photoresponsivity is proportional to the photocurrent (Equation (2)), it follows that the photosensitivity decreased as the film became thicker. To elucidate this observation, the optical properties of the films were evaluated. For reference, it was confirmed that a blank SiO_2_ substrate did not exhibit any photoconductivity.

**Figure 8:**
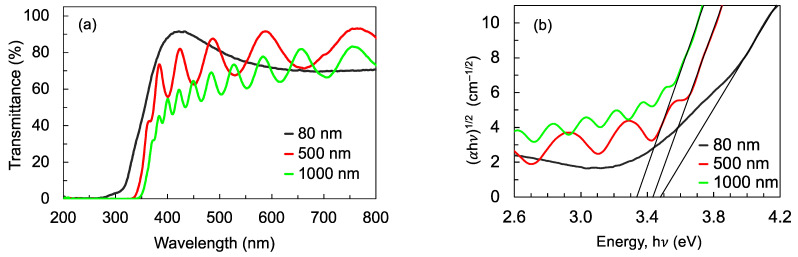
(**a**) Transmission spectrum; (**b**) Tauc plot showing the optical band gap of the TiO_2_ films.

Figure 8a shows the transmission spectra of the TiO_2_ thin films with thicknesses of 80 nm, 500 nm, and 1000 nm. The transparency of the film around the short wavelength end of the spectrum from about 350 nm to 500 nm is observed to decrease as the thickness increased. The transmission edge also shifted to a longer wavelength and correspondingly, the optical band gap decreased, as shown in the Tauc plots in Figure 8b. The decrease in transparency and optical band gap indicates that the number of defects in the film increased as the film became thicker. Oxygen vacancies and trivalent titanium ions are the most common defects formed in TiO_2_ and these defects create deep electron traps, which manifest as localized states that are 0.75–1.00 eV below the conduction band minimum [31,32]. The presence of these localized states effectively decreased the optical band gap as well as the transmittance around the 350 nm to 500 nm wavelength range of the thicker films.

**Figure 9:**
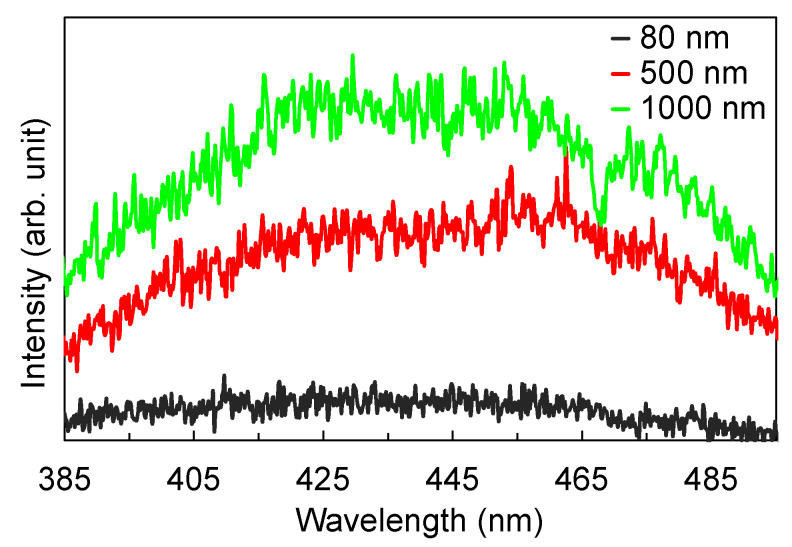
PL emission spectra of the TiO_2_ films. The increase in the PL intensity coincides with the decrease in the transparency of the thicker films.

**Figure 10:**
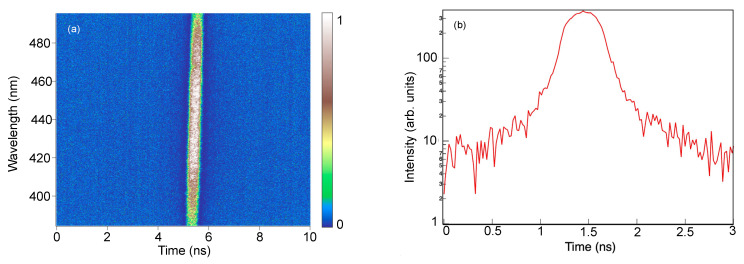
(**a**) Streak camera image showing the time-resolved PL spectrum of TiO_2_ with 1000 nm thickness; (**b**) temporal profile of the time-resolved PL spectrum. The PL emission has a fast decay time of about 2 ns.

The time-independent PL emission spectra of the films were obtained to confirm the nature of the defects observed from the transmission spectra. Figure 9 shows that the films exhibited a broad PL, centered around 445 nm, whose intensity increased as the thickness of the film increased. The wavelength range of the PL emission coincides with the wavelength range in the transmission spectrum where the transmittance was seen to decrease as the thickness of the film increased. Incidentally, as the transmittance decreased, the PL intensity increased. To further ascertain the origin of this PL emission, Figure 10a,b show the time-resolved PL spectra of the film with 1000 nm thickness. The PL emission is extremely fast, with a decay time of about 2 ns. A similar fast decay time was also observed for the films with 80 nm and 500 nm thickness. The fast PL is therefore attributed to radiative recombination of electrons in deep traps and holes in the valence band, modulated by self-trapped exciton recombination. These results confirm that as the thickness of the film increases, the number of defects that serve as electron traps also increases. Consequently, these trapping centers reduces the effective concentration of the photo-generated carriers. The combined effect of the defect traps and the reduced carrier concentration resulted to the decrease in the photocurrent as the thickness of the film increased. Several studies have reported a similar increase in the number of defects and carrier recombination centers with the increase in thickness of metal oxide layers, for example in ZnO [33,34,35], ZnGa_2_O_4_ [36], and TiO_2_/NiO [37].

**Figure 11:**
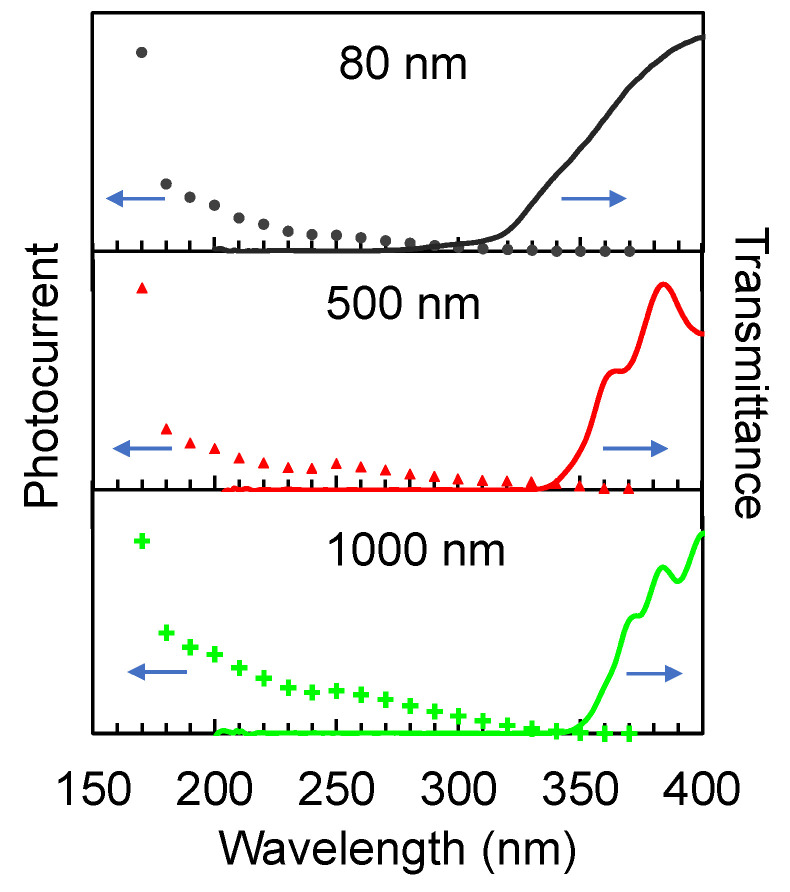
Wavelength response of the TiO_2_ thin film detectors. The cut-off wavelength is shifted to longer wavelength as the film became thicker, in accordance with the red shift in the transmission spectrum.

Figure 11 shows the wavelength response of the detectors, where the photocurrent was measured at each excitation wavelength for a fixed bias voltage of 10 V. The detector with 80 nm film thickness has a cut-off wavelength of about 280 nm, making it responsive to wavelengths that are shorter than this cut-off. As the film becomes thicker, the cut-off shifts to a longer wavelength such that the detector starts to respond at 310 nm (500 nm thick film) and 320 nm (1000 nm thick film) wavelength. The shift in the cut-off wavelength is consistent with the red shift in the transmission spectrum and the decrease in the optical band gap for the thicker films.

**Figure 12:**
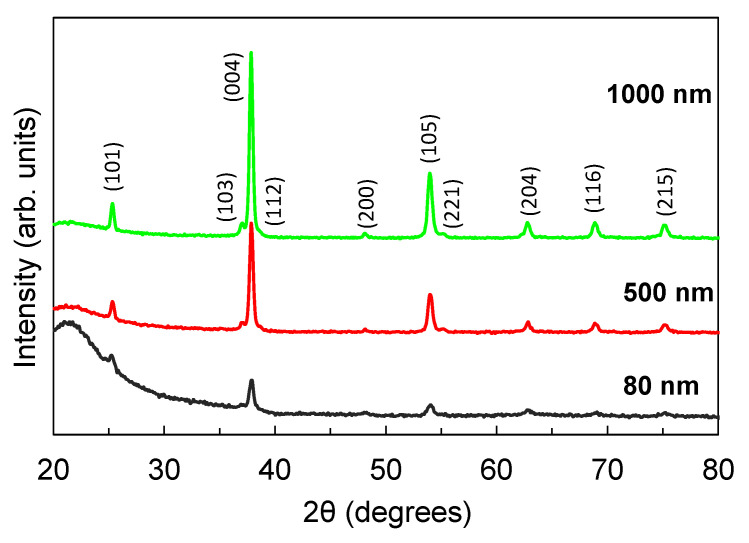
GIXRD spectra for the TiO_2_ films deposited on SiO_2_ substrate with thicknesses of 80 nm, 500 nm, and 1000 nm.

The GIXRD spectra of the films are shown in Figure 12. All films were observed to have an anatase phase and a preferred grain orientation in the (004) direction. The crystallinity index (CI) of the film was calculated using the following:

(4) $$\mathrm{CI}=\frac{A_{c}}{A_{c}+A_{a}}\times 100$$

where *A_c_* is the area under the crystalline peaks and *A_a_* is the area under the amorphous hollows [38,39]. The CI of the 80 nm, 500 nm, and 1000 nm films are 15.3%, 40.6%, and 62.2%, respectively, indicating that the crystallinity improved as the film became thicker. As discussed in the previous section, a high degree of crystallinity contributes to a high photocurrent output. However, the decreased carrier density and mobility arising from the increased number of defects in the thicker films as observed in the transmission and PL spectra appear to have a more significant effect. As a result, the detectors with the thicker film exhibited a lower photocurrent. The 1000 nm thick TiO_2_ film detector contained intrinsically more recombination states, resulting in a higher PL intensity (Figure 9) and lower photocurrent (Figure 6) compared with the 80 nm thick TiO_2_ film detector. A similar behavior was reported in ZnGa_2_O_4_ thin film transistors, whereby the conductivity in ZnGa_2_O_4_ epilayers was influenced more by the number of existing oxygen vacancies than by crystallinity [36].

**Figure 13:**
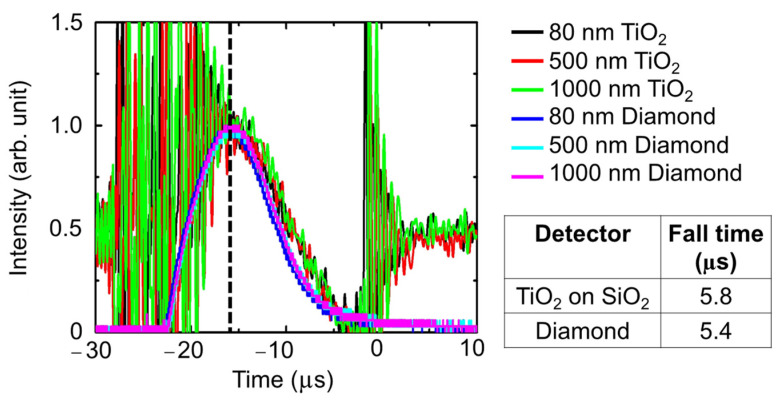
Time response of the TiO_2_ thin film detectors. For comparison, the time response of a commercial diamond UV sensor is also shown.

The response time of the detectors is shown in Figure 13. For comparison, the response time of diamond sensors with thicknesses that are similar to the thickness of the TiO_2_ films was also measured and shown in the same figure. The response time of the TiO_2_ detectors was estimated to be about 5.8 μs, which is within 10% of the 5.4 μs response time of the diamond sensor.

## 4. Conclusions

In conclusion, VUV photoconductive detectors were developed using TiO_2_ thin films deposited on Si, SiO_2_, and SLG substrates at film thicknesses of 80 nm, 500 nm, and 1000 nm. GIXRD spectroscopy showed that the average crystallite size of the 80 nm film deposited on Si is half the average crystallite size of the film in SiO_2_. The much smaller crystallite size decreased the mobility and the lifetime of the photo-generated carriers, resulting in a lower photosensitivity but a faster response time for the detector on Si compared with the detector on SiO_2_. The detector on SiO_2_ exhibited a photosensitivity of 99.99% and a photoresponsivity of 0.44 A/W. The detector on SLG exhibited the worst photosensitivity and photoresponsivity because of its amorphous structure and lower transparency, which allude to the existence of defects that reduce carrier mobility. As the films deposited on SiO_2_ became thicker, the photocurrent decreased. Investigation of the cause of this decrease showed that as the film became thicker, the number of defects that serve as electron traps also increased. Consequently, these trapping centers reduced the effective concentration of the photo-generated carriers, resulting in the decrease in the photocurrent, although the thicker films had better crystallinity. On the other hand, as localized states are created by these defects that effectively decrease the optical band gap of the detectors, the cut-off wavelength of the detector is also shifted to a longer wavelength for the thicker film. The response time of the TiO_2_ detectors on SiO_2_ substrate is within 10% of the 5.4 μs response time of the commercial diamond sensor that was used as reference. The results show that a TiO_2_ film deposited on SiO_2_ substrate with a film thickness of 80 nm exhibit excellent conditions for a photoconductive detector in the VUV region. The development of VUV photoconductive detectors, based on TiO_2_ thin films, will enhance the detection of VUV radiation, benefitting a vast number of industrial and scientific applications. Future work could investigate the temperature- and humidity-dependent photoconductivity characteristics of the detectors.

## Figures and Tables

**Table 1:** Photosensitivity and photoresponsivity of the TiO_2_ thin film detectors at a bias voltage of 70 V.

Substrate	Photocurrent (mA)	Dark Current (mA)	Photosensitivity (%)	Photoresponsivity (A/W)
Si	19.07	0.15	99.19	1.45
SiO_2_	5.35	8.83 × 10^−6^	99.99	0.44
SLG	3.00 × 10^−6^	3.03 × 10^−8^	98.99	1.79 × 10^−7^

## Data Availability

The data presented in this study are available on request from the corresponding author.

## Supplementary Materials

The following are available online at https://www.mdpi.com/article/10.3390/nano12010010/s1, Figure S1: (a) Photocurrent from the TiO_2_ film on Si substrate detector is two orders of magnitude higher compared to the photocurrent from a reference Si substrate; (b) Dark current and photocurrent from a reference Si Substrate are similar.
