# Supplementary material for: Effect of Substrate and Thickness on the Photoconductivity of Nanoparticle Titanium Dioxide Thin Film Vacuum Ultraviolet Photoconductive Detector
Source: Nanomaterials (Basel). 2021 Dec 21;12(1):10. doi: 10.3390/nano12010010 (PMC8746592; doi:10.3390/nano12010010)
Supplement: Supplementary file 1 [file nanomaterials-12-00010-s001.zip › nanomaterials-1467922-supplementary.pdf]

Supplementary Materials

Effect of Substrate and Thickness on the Photoconductivity of Nanoparticle Titanium Dioxide Thin Film Vacuum Ultraviolet Photoconductive Detector

Marilou Cadatal-Raduban [1,2,*], Tomoki Kato [3], Yusuke Horiuchi [3], Jiří Olejníček [4], Michal Kohout [4], Kohei Yamanoi [2] and Shingo Ono [3]

1 Centre for Theoretical Chemistry and Physics, School of Natural and Computational Sciences, Massey University, Auckland 0632, New Zealand
2 Institute of Laser Engineering, Osaka University, 2-6 Yamadaoka, Suita, Osaka 565-0871, Japan; yamanoi-k@ile.osaka-u.ac.jp
3 Department of Physical Science and Engineering, Nagoya Institute of Technology, Nagoya, Aichi 466-8555, Japan; tomoki.kato.onolab@gmail.com (T.K.); masa.horiuchi09@gmail.com (Y.H.); ono.shingo@nitech.ac.jp (S.O.)
4 Department of Low-Temperature Plasma, Institute of Physics, Czech Academy of Sciences, Na Slovance 2, 182 21 Praha 8, Czech Republic; olejn@fzu.cz (J.O.); kohout@fzu.cz (M.K.)
* Correspondence: m.raduban@massey.ac.nz or cadatal-m@ile.osaka-u.ac.jp

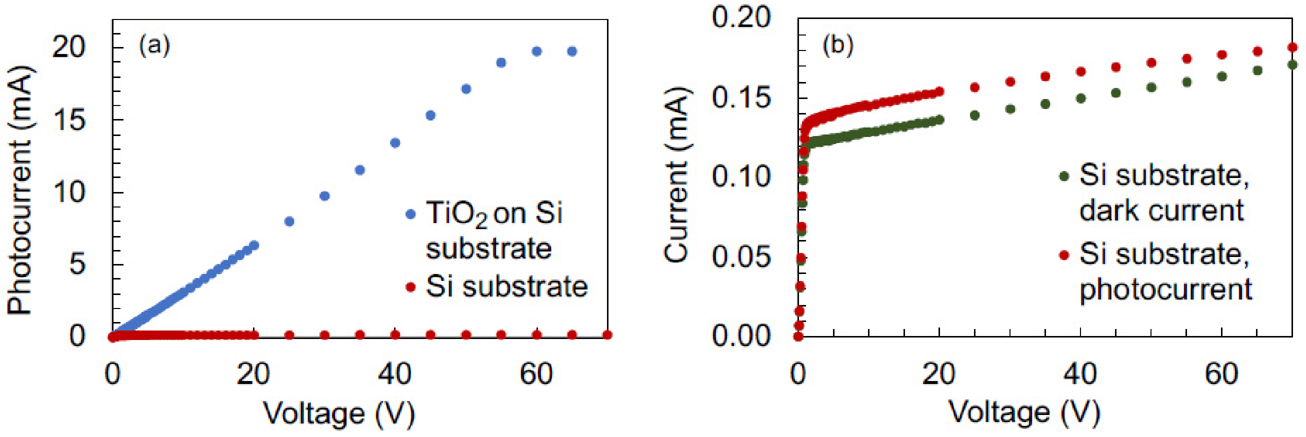

Figure S1. (a) Photocurrent from the TiO2 film on Si substrate detector is two ouders of magnitude higher compared to the photocurrent from a reference Si substrate; (b) Dark current and photocurrent from a reference Si Substrate are similar.

These indicate that the reference Si substrate is non-photoconductiv and that the measured photocurrent is from the TiO_2 film.
